# Mental Fatigue and Sports Performance of Athletes: Theoretical Explanation, Influencing Factors, and Intervention Methods

**DOI:** 10.3390/bs14121125

**Published:** 2024-11-24

**Authors:** Chang-Hong Wu, Yun-Di Zhao, Fu-Qiang Yin, Yang Yi, Lu Geng, Xia Xu

**Affiliations:** 1 School of Sports Medicine, Wuhan Sports University, Wuhan 430079, China; 2021420010@whsu.edu.cn; 2 School of Future Technology, Xi’an Jiaotong University, Xi’an 710049, China; zhaoyd2001@stu.xjtu.edu.cn; 3 Football Academy, Wuhan Sports University, Wuhan 430079, China; 2022410511@whsu.edu.cn; 4 Sports Drug Rehabilitation Center of Physical Education, Southwest University, Chongqing 400715, China; yangyi99@email.swu.edu.cn; 5 Key Laboratory of Exercise Training and Monitoring, Wuhan Sports University, Wuhan 430079, China

**Keywords:** mental fatigue, athlete, sports performance, sport intervention

## Abstract

Mental fatigue is an important factor affecting athletes’ performance. Explaining the effects of mental fatigue on sports performance from a theoretical point of view can help us deeply understand the interconnection between mental fatigue and sports performance and conduct effective interventions based on this. Combining the relevant literature in China and abroad reveals that the current academic theories on the mechanism of sports fatigue include motivational control theory, underload theory, neural waste disposal hypothesis, and resource depletion theory. The effects of mental fatigue on performance are reflected in aerobic endurance, sports decision-making, tactical performance, and technical performance. Current coping strategies for mental fatigue include physiological coping strategies based on nutrition (caffeine), odor, and noninvasive neuromodulation techniques and psychological and behavioral coping strategies based on music and positive thinking.

## 1. Introduction

The continuous development of competitive sports has led to improvements and refinements in the physical fitness and skills of athletes worldwide. However, fatigue is inevitable during sports activities. Prolonged high-intensity high-density training and competition not only affect athletes’ physical health but also produce a sense of fatigue. Various physiological and social problems caused by athletes’ fatigue are becoming increasingly prominent [1].

In contrast to other parameters used in athletic training assessments, fatigue is difficult to quantify using specific data. It can be broadly categorized into two types, motor fatigue, which is a type induced at the physical level, and mental fatigue (MF), which is induced at both the mental and cognitive levels. Both motor and MF have significant impacts on human performance [2]. The concept of MF has attracted a great deal of attention in the last decade [3]. MF is a well-defined concept in daily life. It can occur after limited cognitive work and increases the risk of error in the normal work of mental occupations, such as doctors and nurses in healthcare, precision instrument operators and air traffic controllers in industry, and military personnel [4]. Similarly, in sports, MF can have a significant impact on the quality of performance and presentation of athletes [5]. This study elucidates the impact of MF on athletes’ sporting performance and the strategies employed to address it by synthesizing research on MF and sports performance conducted in China and in international contexts. This study provides theoretical and practical support for enhancing athletes’ sports performances and coaches’ coaching and training proficiencies.

In this narrative review, we aim to provide a qualitative synthesis of the existing literature on mental fatigue in athletes, focusing on the evolution of understanding and the impact on performance. It is important to note that our approach is not that of a systematic review or meta-analysis, which would offer a quantitative synthesis, but rather a thematic exploration of the subject matter.

To ensure a comprehensive exploration of the topic, we conducted a literature search across various databases including PubMed and Web of Science. Our search strategy encompassed the terms ‘mental fatigue’, ‘athletes’, and ‘sports performance’, focusing on studies published from 2000 to the present. This approach allowed us to capture a broad spectrum of qualitative insights into the phenomenon of mental fatigue in athletes

## 2. Theoretical Explanations and Neural Mechanisms

The current academic explanations for MF include four main theories: motivational control theory, underload theory, resource depletion theory, and the neurotoxic waste disposal hypothesis.

The motivational control theory postulates that when the energetic costs associated with a task exceed perceived rewards, MF is the consequence of a diminished willingness to sustain performance due to an imbalance between the effort expended and the rewards received during task performance [6,7]. This theory posits that, during prolonged periods of cognitive performance, the brain’s reward system subconsciously evaluates the costs and benefits associated with sustained task performance. The low- and high-cost outcomes of such analyses are posited to lead to a sense of fatigue, a decrease in motivation or willingness to invest in sustained task performance, and a subsequent decline in task performance [6]. Accordingly, this assertion posits that objective MF resulting from time on task (TOT) may be associated with reduced activity in the motivational and cognitive control regions of the brain [6,7]. Areas of the cognitive control network include the anterior cingulate cortex, dorsolateral prefrontal cortex, and dorsolateral premotor cortex [8]. Studies have found that while TOT results in reduced activity in some areas, brain areas that provide the motivational drive for behavior, including the ventral midbrain and ventral striatum, are unaffected by TOT [6]. Similarly, the subjective experience of MF was not associated with activity in the brain’s motivational circuitry [9]. Consequently, reduced motivation is unlikely to be an explanatory mechanism for MF.

The underload theory provides an alternative explanation for the subjective perception of fatigue and reduced task performance during sustained or prolonged cognitive task performance. This theory proposes that prolonged performance of inherently simple and repetitive cognitive tasks may cause participants to experience boredom, which leads to a negative effect, decreased task engagement, and increased task-irrelevant thoughts, resulting in decreased cognitive performance [10,11]. As TOT increases, increased activity in the default mode network (DMN) structure may cause participants to increase their level of task-irrelevant thought activity, leading to an increased frequency of attentional lapses and decreased levels of cognitive performance. The DMN becomes active when the brain is in a state of wakefulness and rest or when the individual is engaged in an internally directed thought unrelated to an externally directed goal-directed task. The DMN is the brain system that becomes active when the brain is at rest or when the individual is engaged in internally directed thoughts unrelated to externally driven goal-directed tasks [12]. Structures that make up the DMN include the posterior cingulate cortex, posterior spinal cortex, medial prefrontal cortex, precuneus, and subparietal lobule. Increased activity in these structures is negatively correlated with activity in the cognitive control network (CCN), suggesting their potential role in reducing cognitive control in goal-directed tasks [13]. Studies have suggested that inactivation of the CCN may be associated with increased activity in the DMN, as activity in both networks is negatively correlated [12], and that the subjective experience of fatigue is correlated with the experience of boredom on the basis of the underload theory. That is, studies posit a positive correlation between DMN activity and the subjective experience of fatigue. However, existing research suggests a negative correlation between DMN activity and subjective fatigue [9]. Therefore, the underload theory does not seem to be a reasonable explanation for MF. As a negative correlation exists between MF and the subjective experience of brain fatigue induced by TOT and DMN partial activity, the negative correlation between reduced activity in the cognitive control regions of the brain and DMN partial activity may be explained in terms of the other two theories of MF discourse.

The resource depletion theory suggests that the decline in subjective experience and objective performance in MF is the result of the depletion of available cognitive resources due to the continued allocation of attention to the task [14,15,16]. According to this hypothesis, the observed deactivation of the anterior cingulate cortex, dorsolateral prefrontal lobe, dorsolateral premotor cortex, and anterior supplementary motor area may be due to the gradual depletion and overuse of available metabolic resources in these areas. These regions form cognitive control networks that support the top-down control of behavior and are responsible for the brain’s executive functions, including working memory, response inhibition, planning, cognitive flexibility, and shifting and maintaining the focus of attention [8,17,18]. As the MF develops primarily as a result of sustained attention to cognitive tasks that require some degree of executive control [19], the TOT-induced reduction in activity in these brain regions can be interpreted as a functional change caused by metabolic mechanisms associated with resource depletion [20]. Sustained attention to a cognitive task is an executive function. According to this theory, the prolonged use of these sub-resources over time may lead to the development of MF. According to existing evidence and pre-meta-analyses, sustained attention is mainly supported by right-lateralized cortical and subcortical structures [10]. However, in addition to criticism of the lack of rigor in the statistical underpinnings of the TOT theory [21,22], another inherent obstacle to its acceptance is disagreement over what constitutes a cognitive resource that is depleted by TOT. Researchers have argued that blood glucose is a cognitive resource that is likely to be depleted, which has not yet been supported by sufficient experimental data [9].

Finally, the neurotoxic waste disposal hypothesis suggests that objective and subjective MF does not result from the depletion of resources in a task but rather that the continued use of cognitive resources leads to the accumulation of toxic waste in neural tissues. Activity in the cognitive control network is consequently reduced to reduce the accumulation of neurotoxic waste, maintain homeostasis in the body, and minimize potential damage to the brain tissue [9]. Specifically, the brain regions responsible for cognitive control, particularly the anterior cingulate cortex, produce large amounts of amyloid beta, a neurotoxic substance that can damage the brain during activity. This, in turn, activates a negative feedback loop that slows down its production to keep the brain safe. According to this hypothesis, the rate of amyloid-beta accumulation in an MF task corresponds in part to the sense of effort required to complete the task, and the sense of MF resulting from the completion of a cognitive task depends fundamentally on the absolute amount of amyloid-beta in the brain [9]. However, as the mechanisms involved in this theory are at the physiological level, further experimental evidence is required (Table 1).

Neurotoxic waste accumulation theory posits that sustained cognitive activity leads to the accumulation of neurotoxic waste products, such as beta-amyloid (Aβ) peptides, in neural tissues. This accumulation is proposed to decrease activity in cognitive control networks to mitigate potential brain damage and maintain homeostasis, aligning with the “waste disposal hypothesis” of mental fatigue as detailed by Holroyd [9]. Previous studies have indicated a potential link between beta-amyloid accumulation and mental fatigue. Specifically, the rate of Aβ accumulation during mentally fatiguing tasks has been associated with the sensation of effort required for task performance, and the feeling of mental fatigue is thought to be determined by the absolute amount of Aβ in the brain [23], which is in accordance with the findings of Selkoe [24] on the physiological production of β-amyloid protein and its role in Alzheimer’s disease. Additionally, we have included the positive effects of sleep on mental fatigue recovery, as sleep is known to clear the neural system of accumulated Aβ peptides [25].

**Figure 1:**
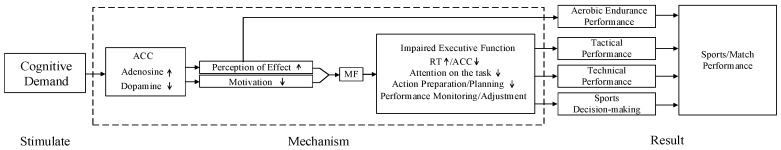
Conceptual model of the resource depletion theory for MF.

The above four theories provide explanations for MF from different perspectives, although they all have different degrees of shortcomings, including the resource depletion theory and waste disposal hypothesis, which need to be supported by more experimental results. However, some researchers have proposed a conceptual model of MF based on the resource depletion theory [26], which seems to better understand the mechanisms by which MF negatively affects task or sport performance (Figure 1).

The motivational control theory posits that MF results from a diminished willingness to sustain performance due to an imbalance between the energetic costs and perceived rewards of a task. However, research has found that the relationship between the motivationally driven brain regions and the subjective experience of MF with time on task (TOT) is not as expected by the theory, suggesting that reduced motivation is unlikely to be the main explanatory mechanism for MF. The underload theory proposes that prolonged performance of simple and repetitive tasks leads to boredom, negative sensorimotor qualities, reduced task engagement, and increased task-irrelevant thoughts, resulting in decreased cognitive performance. Nevertheless, the correlation between default mode network (DMN) activity and subjective fatigue in this theory is inconsistent with existing research findings, indicating that it is not a reasonable explanation for MF either. The resource depletion theory states that MF is caused by the depletion of available cognitive resources due to the continuous allocation of attention. Although it has some plausibility, there is disagreement about the cognitive resources depleted by TOT and the rigor of its statistical basis has been questioned. The neurotoxic waste disposal hypothesis suggests that MF is due to the accumulation of toxic waste in neural tissues caused by the continuous use of cognitive resources to reduce neurotoxic waste accumulation. However, this theory involves physiological mechanisms and currently lacks sufficient experimental evidence to support it.

In conclusion, these four theories explain MF from different perspectives, each with different degrees of shortcomings, and no single theory can fully explain MF. The occurrence of MF may be the result of multiple factors, and its complexity requires a comprehensive consideration of different theoretical viewpoints for in-depth understanding.

Despite the strong association between MF and negative outcomes, the neural mechanisms underlying subjective negative experiences and reduced performance when individuals experience MF remain unclear [27]. In a narrative review of the neural mechanisms of MF, researchers proposed a dual regulatory system consisting of neural facilitatory and inhibitory systems to explain the mechanisms behind acute MF [28]. They proposed that the cortico-striatal-thalamo-cortical loops connecting the limbic system, basal ganglia, thalamus, and frontal cortex form a psycho-promoting system. Moreover, motivational inputs from mental tasks and stimulation of this system prevent a decline in task performance, and a psycho-inhibitory system is thought to be related to the insula and posterior cingulate cortex. The stimulation of this system during mental tasks leads to a decline in cognitive performance. Thus, acute MF may result from unconscious activation of the mental facilitation system, enhancement of the mental inhibition system, or a combination of both [9]. Although this study provides a mechanism for further elucidation of the mechanisms associated with MF, it is only a narrative review that builds on previous research and does not include a systematic literature search. In addition, this study did not include functional imaging studies and did not assess changes in brain activity throughout the duration of mental task performance. Thus, it did not provide any insights into the extent to which MF develops subjectively and objectively or the regulation of activity in brain regions during task performance. Other perspectives on the mechanisms of MF suggest that acute subjective and objective MF develop as a result of long-term cognitive performance, leading to a reduced ability to sustain task performance, possibly because of resource depletion, accumulation of neurotoxic waste products, or boredom among other reasons [11].

Some studies related to MF imaging suggest that a novel approach to inducing MF using a specific cognitive task is to simultaneously monitor the progression of brain activity, cognitive task performance, and subjective sensations of MF as the fatiguing task progresses over time levels [29]. Thus, changes in brain activity associated with objective and subjective measures of MF can be investigated, and the underlying neural mechanisms can be explained.

## 3. The Effect of MF on Athletic Performance

### 3.1. Endurance Performance

Endurance is a very important physical ability in many sports, and its importance for athletes in these sports cannot be underestimated. According to previous studies, the negative impact of mental fatigue on endurance performance is undeniable. A meta-analysis showed that despite significant individual differences in susceptibility to MF and gender, age, BMI, and fitness level not being affected by MF (possibly due to methodological differences), the negative effect of MF on whole-body endurance was generally confirmed [30]. Some researchers have suggested that MF has a greater effect on aerobic endurance than on anaerobic endurance in athletes [2]. A study on the specialized skills of Australian amateur footballers showed that MF affected aerobic endurance performance [31]; however, whether MF has a negative effect on endurance cannot be definitely concluded yet. A study of MF on endurance performance in high-temperature conditions from an environmental perspective showed that mild MF was not sufficient to significantly alter performance in subsequent endurance tasks at a relatively high temperature of 30 °C and that athletes who regularly trained for endurance were able to resist the negative effects of mild MF and subsequent endurance performance to some extent [32]. This suggests that the effect of the MF on endurance performance is limited by its degree.

Individual motivation has also been implicated in the effect of MF on endurance. A meta-analysis showed that, while the duration and intensity of physical activity were important factors in the reduction in physical performance by MF, increased perceived exertion was the most important source of the negative effects of MF on endurance [2]. Another study found that under high cognitive load conditions, MF increased perceived exertion while impairing physical endurance performance, whereas monetary incentives counteracted the MF-induced decrease in endurance performance [33]. Thus, MF influences endurance performance through two pathways: perceived effort (“I can’t do it, I’m too tired”) and reward value (“I don’t want to do it, it’s not worth it”). Thus, rational regulation and manipulation of athletes’ intrinsic motivation and reward value can help athletes resist MF and improve endurance performance [34]. However, according to the Westerners’ effect, the intervention of an external motivator, such as monetary incentives, can undermine an individual’s intrinsic motivation; therefore, caution should be exercised when manipulating it.

One study proposed the adenosine hypothesis to explain the physiological mechanism by which MF impairs endurance performance. During MF, adenosine accumulates in the anterior cingulate cortex (ACC) and right superior frontal lobe (SFL). Due to the antagonistic effect of adenosine on dopamine, adenosine accumulation leads to a decrease in dopamine; large amounts of adenosine increase perceived effort during endurance performance, and a decrease in dopamine decreases motivation to exert effort. High levels of adenosine increase the perceived exertion of endurance performance, whereas a decrease in dopamine decreases the motivation to exert effort. This ultimately leads to a reduction in endurance performance due to a combination of factors [35]. This hypothesis has been supported [3].

Current research suggests that physiological parameters closely associated with endurance exercise, such as respiration, heart rate, cardiovascular metabolism, lactate production, and neuromuscular function, do not appear to be affected by MF [32,35]. This suggests that the relationship between MF and endurance cannot be explained by the various physiological factors mentioned above. Therefore, the management of MF from the perspective of improving endurance can be approached from the perspective of increasing brain neurotransmitter concentrations to improve athletes’ drive as well as considering the use of specific training methods. One study found that brain endurance training (BET) prior to formal physical training could improve endurance performance in athletes to a greater extent than physical training alone [36].

### 3.2. Sports Decision-Making

Decision-making is the process of choosing between a range of alternatives for an actual program [37]. Sports decision-making is a complex cognitive process that involves a variety of skills, including an athlete’s ability to extract and process cues from the external environment. To achieve better decision-making performance, athletes need to improve their perceptual-action coupling abilities to better match their behaviors in time and space. During this process, athletes’ attention, inhibitory control, and cognitive flexibility are closely related to sports decision-making [38]. Sports decision-making is one of the most important aspects of athlete performance, especially in team sports, where motor decisions such as passing, shooting, and tactical running are particularly important [39]. Therefore, the first step is to identify behaviors that can be performed according to an athlete’s ability. For instance, in ball sports, athletes need to make accurate and effective judgments about the trajectory of the ball to control it, and in team sports, athletes need to consider whether their teammates are in a better scoring position than they are to make the best decisions.

Currently, almost all studies in the field of sports about MF on sports decision-making show its negative effects. Studies have suggested that MF can lead to football players being unable to respond in a timely manner to changes in their teammates’ positions, reducing the accuracy of football-specific sports decision-making, reducing shooters’ decision-making regarding the correct target to shoot at (unchanged shooting accuracy, with an increased probability of hitting the wrong target), and decreasing overall scoring tendencies in cricket-specific sports [39]. As most sports emphasize the need for athletes to quickly identify and interpret relevant cues and develop appropriate responses, as well as to use these perceptual skills to decide on appropriate technical inputs and movements with reference to the external environment and cues, superior perceptual skills are required for decision-making in sports. However, MF impairs athletes’ relevant perceptual and cognitive performance, which, in turn, impairs sports decision-making [26]. In terms of behavioral performance, the effect of MF on sport decision-making does not impair the accuracy of passing decisions in ball sports [40,41,42] or increase the reaction time of sports decisions [39,42]. However, owing to certain differences between sports, the effects of MF on sports decision-making in different specialties are also somewhat different. Current academic research on the effects of MF on sports decision-making is mostly found in football (including small-field games and normal games), basketball, and other ball games. Studies have indicated that MF can have a negative impact on sports decision-making, such as dribbling, passing, shooting, or kicking [37,40,41,43,44], as well as on attacking and defending decisions [23].

In team sports, athletes need to pay attention to their teammates and opponents; however, MF causes athletes to focus excessively on their opponents and not on their teammates’ actions [40,41]. Some studies have suggested that MF diminishes athletes’ capacity to perceive environmental cues, leading to alterations in attentional and decision-making strategies, and consequently affecting the distribution of gaze times between opponents and teammates. [37]. Moreover, MF causes players to use less relevant information to influence their decision-making process [44]. Due to the reduced information involved in decision analysis (e.g., positional information of teammates and opponents) and altered latent perception, players are more likely to make poor decisions; for example, a player may decide to pass his opponent in the presence of MF; however, this pass does not necessarily create more scoring chances for teammates, a decision the player would not have made in the absence of MF.

The negative effect of MF on motor decision-making is essentially the impairment of executive function by prolonged cognitive tasks. MF has variable effects on neural pathways related to motor decision-making in the anterior cingulate cortex, dorsolateral prefrontal lobe, anterior supplementary motor area, frontal inferior frontal gyrus, and medial superior parietal lobe, with the anterior cingulate cortex region being most affected by MF [2]. Although studies have mechanistically suggested that MF negatively affects brain regions associated with cognitive performance, which in turn impairs motor decision-making, cognitive performance does not always decline when MF occurs due to certain compensatory effects in the body.

### 3.3. Tactical Performances

Tactics is one of the most important aspects that athletes need to think, evaluate, and make decisions about when playing sports. Tactics have different manifestations in different sports, especially in some antagonistic same-field sports, where tactical performance is crucial to winning or losing the game. Currently, few studies exist on the effect of MF on tactical performance, and all of them focus on football. In football, the level of teamwork among team members and the speed at which the team disperses and contracts can be used to examine players’ tactical performance. This requires the ability to quickly identify information about the environment and use it to formulate strategies to guide their motor behavior. Some studies have investigated this using methods such as the Team Stretch Index (TSI) or Spatial Exploration Index (SEI) [45]. A study found that MF harms the way small-field football players engage with their surroundings, as well as their positional decision-making, team spread, and spatial exploration. For instance, it lessens players’ lateral synchrony. This affects their capacity to sense environmental information and keep making sound decisions, causing shifts in positional details. Consequently, players’ ability to obtain information from their teammates and opponents is weakened. Compared with the control group, the MF group showed a more significant decrease in team contraction speed and a higher-than-average dispersion speed, suggesting that the fatigue effect induced by MF decreases the ability of players to collect and use available information [45].

Another study confirmed that MF causes a significant reduction in synchronous interactions between teammates in small-field games and impairs the ability to appropriately monitor and adjust the athletic performance of the position in which they are placed. Moreover, it indicates that players in a state of MF use inappropriate information to make decisions about their position, which leads to an inability to correctly identify and use available spatial information on the court [46]. Furthermore, the study noted that MF caused players to run shorter distances at medium and high speeds, which also suggests that MF impairs high-intensity athleticism, affecting players’ ability to execute tactics. Another study indicated that MF affects players’ peripheral vision perception, which constrains the frequency of executing penetration, deep movement, and uniform defensive actions at the tactical behavioral level. Specifically, MF restricts the frequency of attacking tactical actions, including dribbling toward the opposing team’s goal, and affects the defenders’ defensive strategies in the last row. In conclusion, under the effect of an MF, football players show less efficient tactical behavior and a higher rate of tactical errors [47].

To provide a concrete example, a study by Trecroci et al. (2020) investigated the effects of mental fatigue on physical activity, technical performance, and decision-making performance during small-sided games among sub-elite soccer players. The findings demonstrated that mental fatigue impaired the distance covered in acceleration per minute, increased negative passes, and decreased passing and shot accuracy compared to a control task after the mental fatiguing protocol. Decision-making performance in negative passes, pass accuracy, and dribbling accuracy also likely decreased. These results underscore the detrimental effects of mental fatigue on the technical and decision-making aspects of soccer-specific performance, which aligns with our discussion on the impact of MF on ball control and precision [44].

A recent meta-analysis showed that MF had no significant effect on the total running distance and tactical behavior of footballers in small-field games [48]. However, the results of this study, which were limited in scope (based on six research trials) and small in sample size (sample size of 75), need to be treated with caution; therefore, the results that MF has no significant effect on the total running distance and tactical behavior of football players during small field games cannot be generalized.

### 3.4. Technical Performance

Different groups and specialized sports have different technical requirements. Research on the effects of MF on technical performance has been conducted in a number of specialist areas, such as football, basketball, table tennis, cycling, swimming, canoeing, badminton, and competitive gymnastics, with the largest number of studies in football. A review of these studies has shown that MF alters the speed-accuracy trade-off in sports, thus affecting technical performance in sports such as boxing and fencing. Moreover, MF negatively affects technical performance in lower limb-dominant and upper limb-dominant sports, such as passing accuracy, interception rate, shot speed, and shot accuracy in football and ball speed and ball accuracy in table tennis. Accordingly, the researcher hypothesized that free throws in basketball, drop-kick shooting in rugby, and throwing in darts would be affected by the MF [39]. Another systematic review of the effect of MF on the technical performance of athletes found that the negative effects of MF on motor performance, in terms of decreased accuracy and increased execution time, may be due to the fact that MF impairs executive function [23]. However, the conclusion that MF has a greater effect on offensive skills than on defensive skills is not conclusive in this study, as seven studies on offensive skills were analyzed in this study, compared to only two studies on defensive skills. This suggests that further attention is required with regard to this area to add empirical studies from various perspectives. However, at this stage, the conclusions drawn are often not meaningful due to the small amount of the literature included.

Current academic researchers have conducted more football-specific studies in this area, and most have used football games as specialized vehicles. One study showed that MF negatively affected the offensive and defensive skills of players in small-sided games, mainly in terms of lower positive involvement, ball possession, passing accuracy, tackling, and ball control errors [49]. Furthermore, another study by the team showed that MF affects players’ technical performance in the Loughborough Soccer Passing Test (LSPT) and Shot Test (LSST), resulting in a reduction in both the speed and accuracy of their shots, as well as an increase in the time taken to take a penalty. This was because MF affected player running during the tests, with shorter distances during intermittent running, more errors in passing and ball control, slower shooting speeds, and reduced accuracy, leading to a reduction in passing and shooting performance [50]. Another review indicated that MF adversely affects the use of fundamental motor skills (FMS). Translating this to specific football skills, MF impairs the performance of offensive and defensive football skills [26]. In addition, MF affects not only ball control, passing, and shooting accuracy but also the number of return passes and distance accelerated per minute, resulting in lower ball control, shanking, and breakaway success and a higher rate of turnover in possession [44,50]. Existing studies on the effect of MF on football-specific technical performance mainly focus on two main aspects of offensive and defensive technical performance: ball control rate, ball control error rate, passing accuracy, passing error rate, stealing success rate, shooting accuracy, shooting speed, penalty time, number of return passes, and intermittent running distance of players. Although a recent systematic review and meta-analysis showed that MF resulted in significant differences in terms of turnover, number of breaks, and rush success compared to non-MF conditions, the available evidence does not support the idea that MF has an impact on passing skills [51]. The review included only eight studies in the literature and only three studies in the passing skills subgroup; therefore, the conclusions drawn are not very informative and should be treated with caution. In addition to affecting some of the technical indicators of football-specific performance mentioned above, MF reduces the athlete’s field of vision, thus prioritizing more direct technical movements in the attacking phase and forcing the athlete to make mistakes in generating passing routes [47].

A narrative review discussing the impact of mental fatigue (MF) on physical, technical, and tactical performance in ball sports indicates that, in addition to the aforementioned negative effects of MF on soccer players in small-sided games, such as reduced participation, increased passing errors, easier loss of ball control, and lower success rate in dispossessing the ball, it also leads to an increase in turnover for basketball players. MF can change the tactical behavior of athletes in team sports (such as neglecting defensive principles and reducing collective tactical behavior) and affect the perception of on-field positions [52]. The reason is that MF has been proven to impair perceptual-cognitive performance and the ability to utilize environmental information and player positioning. Combined with the impaired decision-making ability under MF, this may manifest as athletes making suboptimal strategic choices at critical moments, thereby potentially exposing themselves to a higher probability of injury. This part mainly elaborates on the negative impact of MF on athletes’ tactical decision-making, utilization of environmental information, and strategic choices at critical moments, which in turn affects tactical performance [53]. In the MF state, the speed and accuracy of players’ brain information processing decline, thus affecting their performance in tactical execution. For example, in defense, players need to constantly pay attention to various aspects of information such as the running positions of offensive players and the transfer of the ball. MF may prevent players from making timely and correct adjustments to their defensive positions. In high-intensity team sports, tactical execution often requires rapid decision-making and high-intensity movement coordination within a short time. For instance, in fast-break tactics in basketball games and spiking and blocking combinations in volleyball games, if affected by MF, athletes may make mistakes in executing tactics, such as inaccurate passes and incorrect timing of attacks. However, due to the limitations of current research, the specific degree of influence and mechanisms still need further exploration [54].

Another study on the effects of MF on the technical performance of young footballers of different ages (U14, U16, and U18) showed that impaired passing performance was only observed in the U18 group, but not in the other two groups, suggesting that age does not play a moderating role in the effects of MF [55].

In terms of other specialized technical performances, MF negatively affected paddling efficiency, paddling speed, and time in junior kayakers [56]; reduced swimming speed in junior swimmers [57]; affected ball speed when hitting a table tennis ball; reduced stroke and landing accuracy; increased the number of errors made by players; altered the speed-accuracy trade-off (favoring ball speed over accuracy) of players [58]; increased the number of falls from the balance beam in female gymnasts [59]; and impaired visuomotor reaction time in badminton players [60]. Several studies have shown that MF has a negative effect on technical performance in several sports such as canoeing, swimming, table tennis, badminton, and gymnastics, whereas studies on table tennis have shown a negative effect of MF on hand dexterity.

However, several studies on cycling have shown that the effect of MF may not be significant in all sports. One study comparing the technical performance of professional and amateur cyclists under MF found that professional athletes performed better than amateur athletes under MF conditions, suggesting that professional athletes have better resistance to MF [61]. One study showed that MF did not affect the performance of amateur athletes in cycling speed training, and another study indicated that MF did not affect the performance of amateur athletes in cycling speed training [62]. Another study revealed that MF may affect the endurance performance of sub-elite cyclists, which in turn affects their finishing times [63]. The reason for this difference may be that cycling is a closed sport compared with other disciplines, and MF clearly has a greater effect in open sports (e.g., basketball) than in closed sports. A systematic review of MF in basketball performance showed that MF affects free-throw and three-point shooting rates [64], whereas another study examining the effects of MF on the technical performance of adolescent players found that MF modulates individual endocrine and autonomic responses, including attenuating salivary testosterone concentrations and increasing α-amylase concentrations [65]. Decreased salivary testosterone concentrations may affect dopamine messaging in brain regions associated with cognitive control, ultimately leading to an increase in overall player turnover, thereby affecting the technical performance of basketball players.

## 4. Interventions for MF

### 4.1. Physiological Coping Strategies

The main physiological coping strategies used to manage the negative effects of MF include nutritional interventions, odor interventions, and noninvasive neuromodulation techniques. The most common nutritional intervention administered was caffeine. Caffeine is a natural xanthine alkaloid that positively alters the central nervous system function. Adenosine inhibits the release of excitatory neurotransmitters (e.g., dopamine) in the brain, reducing alertness and spontaneous behavioral activity, and caffeine can easily cross the blood–brain barrier and occupy adenosine receptors in the CNS, preventing them from binding to adenosine to increase neuronal activity and CNS excitability [66]. Thus, intake can be considered a strategy to combat MF. One study showed that caffeine ingestion increased endurance performance in men in the MF state, accompanied by an improvement in the psychological state [66]. Another study demonstrated that caffeine increased athletic performance and emotional arousal in cyclists in the MF state and decreased MF-induced negative feelings and the Rating of Perceived Exertion (RPE). A further study showed that caffeine increased MF-induced negative feelings and RPE. Another study indicated that caffeine increased MF-induced negative feelings and RPE in cyclists in the MF state. RPE) [67]. Moreover, pre-game caffeine intakes of 3–6 mg/kg significantly improved basketball players’ performance in MF susceptible areas, such as vertical jump height, running speed without the ball, tactical improvisation, consistency of three free throws, rebounds and assists, and physical play in specific tests and games [68]. In addition, studies have used a mouthwash made of a mixture of caffeine and maltodextrin, which was also effective in reducing the error rate in athletes [59]. However, as caffeine intake and duration of competition can cause insomnia, which can have a negative impact on athletes’ subsequent competitions, training, or normal life, such strategies need to be carefully considered by coaches and athletes to weigh their advantages and disadvantages.

Receptor-specific recognition present in the human oral and nasal cavities and brain activation that accompanies receptor recognition plays an important role in counteracting the potential for MF. Studies have suggested that synthesizing a mixture containing methyl-naphthylketone, 2-methyl-5-(1-methylethenyl)-2-cyclohexanone (I-carvone), 12-dimethoxy-4-(propen-en-1-yl)benzene (methylisoeugenol), and ethyl phenylacetate with a floral odor similar to that of honey could activate certain olfactory receptors and that the activation of these receptors is effective in reducing the effects of MF [69]. Studies have also proposed that the olfactory bulb is connected to the amygdala–hippocampal complex and that the amygdala plays an important role in the value of rewards associated with cognitive effort. This makes decisions based on the assessment of rewards and potential costs projected to the anterior cingulate cortex. Intermittent exposure to several neutral pleasant odors (e.g., citral and menthol) leads to positive feelings that mitigate the effects of MF on cognitive performance [70], highlighting the importance of positive affect and affective valence as mediating mechanisms through which MF counteracts odor characteristics.

Non-invasive neuromodulation techniques include neurofeedback training, transcranial ultrasound stimulation (tUS), transcranial electrical stimulation (tES), transcranial magnetic stimulation (TMS), and photobiomodulation (tPBM), of which TMS and tES are thought to have beneficial effects on the MF through relevant physiological mechanisms [70]. One study used transcranial direct current stimulation (tDCS) with an anode placed at the Fp1 site of the brain and a cathode placed at the Fp2 site to stimulate the prefrontal brain regions of amateur female swimmers using a protocol of 2 mA DC stimulation for 30 min. This study found that the MF group after tDCS intervention was able to counteract the detrimental cognitive effects and maintain endurance performance [71]. Another study explored the effects of tDCS on MF and physical performance in the left dorsolateral prefrontal cortex (DLPFC) of professional swimmers and found that anodal tDCS to the left DLPFC attenuated the negative effects of MF on 50 m swim performance. Conversely, cathodal stimulation had no significant effect on 50 m swim performance [72]. Current research suggests that tDCS applied to the DLPFC appears to modulate the body’s arousal state and may be a more beneficial coping strategy for MF than caffeine, with fewer side effects [73]. The existing research demonstrates that properly applied anodal tDCS intervention can improve cognitive performance, thereby helping athletes perform better. Specifically, it has the potential to improve mental performance by enhancing and strengthening executive function skills, managing negative emotions such as anxiety and depression, and counteracting MF [74].

### 4.2. Psychological and Behavioural Coping Strategies

Music- and mindfulness-based interventions (MBIs) are common psychological and behavioral strategies for managing MF. Listening to relaxing music can help people feel less mentally fatigued and remain productive, alleviate the MF associated with performing sustained cognitive-motor tasks, and reduce deterioration in motor performance [75]. The role of relaxing music in alleviating MF may explain why people listen to music while working, studying, or driving in their daily lives. Furthermore, educational interventions have shown potential in enhancing awareness and resilience regarding mental fatigue. Mallia et al. (2020) conducted a media literacy intervention focused on Performance and Appearance Enhancing Substances (PAES) use among sport science students, providing novel insights into the management of mental fatigue in athletes [76].

Mindfulness interventions have gained increasing attention as psychotherapeutic approaches to sports. Mindfulness requires participants to observe all experiences as they arise with acceptance, openness, and autonomous willingness rather than impulsively trying to change or avoid them, even if they are unpleasant or unwanted. Mindfulness encompasses a variety of meditative practices, such as breathing and body scanning, the essential elements of which are focused on the present moment, non-judgmental awareness of the present, and receptivity. Mindfulness is a systematic behavioral therapy that provides training in self-regulation and the ability to control one’s behavior; it is defined as the ability to overcome impulses and habitual responses, such as bad emotions or irrational thoughts. Therefore, mindfulness may be a useful strategy for recovery from MF [70]. A study comparing the effects of music training and mindfulness interventions on the recovery of elite volleyball players with MF showed that mindfulness-based training was more effective in reducing post-competition-induced MF than music training; however, the effects of music and mindfulness on recovery from physical fatigue were not observed [77]. Another study examining the effects of a mindfulness intervention on cognitive function in football players revealed the potential benefits of mindfulness training on reaction time and accuracy. Athletes’ cognitive performance (working memory) improved after mindfulness training compared to pre-tests, and the results of a functional near-infrared spectroscopy (fNIRS) study demonstrated significant changes in oxygenated hemoglobin concentrations in specific brain regions after a mindfulness intervention. The results of a functional near-infrared spectroscopy (fNIRS) study identified significant changes in oxygenated hemoglobin concentrations in specific brain regions following mindfulness intervention, and a brief session of mindfulness training also provided useful evidence of a reduction in MF levels and salivary cortisol concentrations after a half-time football match. Therefore, this study recommends the use of specific mindfulness interventions during football matches [78]. This rationale is further substantiated by the structured educational programs targeting mental fatigue management, as highlighted by Mallia et al. (2020), which underscore the importance of media literacy in athletic performance and well-being [76]. A meta-analysis of 32 studies found that athletes showed significant improvements in all indicators of athletic performance after mindfulness interventions [79]. Another systematic review found that a mindfulness intervention directly attenuated MF and had a positive effect on athletes’ attention, aggression, recovery from mind-wandering, and athletic performance (grabbing, planks, basketball free throws, etc.); moreover, emotional inhibition improved more after a mindfulness intervention among men than among women [80]. This gender difference may be because men experience positive emotions more often than women, whereas women experience negative emotions more often, resulting in more stable emotional inhibition in men after mindfulness training.

## 5. Limitations and Prospects

While our narrative review provides a qualitative synthesis of the current understanding of mental fatigue (MF) in athletes, we acknowledge the inherent limitations due to our non-systematic approach in selecting and reviewing the literature. This approach may have influenced the breadth and depth of our synthesis, as compared to a systematic review method that ensures a more rigorous and unbiased assessment of the impact of MF on athletic performance. We concur with the suggestion that future research should employ systematic review methods to enhance the comprehensiveness and reliability of the findings in this domain. Furthermore, our review has primarily focused on the application of tDCS in the context of sports performance. We recognize the potential of other neuromodulatory techniques, such as TMS and tPBM, which warrant exploration for their efficacy in mitigating mental fatigue and enhancing athletic capabilities. The exploration of these alternatives could provide a broader understanding of interventions to address MF and should be considered in future studies.

We also acknowledge that the current review may not have captured all of the relevant studies due to the limitations in our literature search strategy. This could have led to omissions or issues in self-consistency. To address this, we recommend that future research applies more stringent inclusion and exclusion criteria and provides a clear methodology for literature selection, thereby reducing potential biases and ensuring a more representative overview of the field. Lastly, while we have discussed various physiological, psychological, and behavioral coping strategies for MF, we agree that there is a need for further exploration of non-invasive neuromodulation techniques, such as tPBM, in the treatment of MF. These techniques may offer unique characteristics and limitations that could be beneficial in specific sports scenarios.

In conclusion, we have taken the opportunity to clarify and expand upon the limitations of our study, with the aim of providing a more transparent and comprehensive understanding of the current state of knowledge on mental fatigue in athletes. We believe that these revisions will guide future research in addressing the gaps identified and in adopting a more systematic approach to understanding and managing mental fatigue in sports.

In reflecting on the narrative review, it is evident that our approach has allowed us to capture the qualitative trends and patterns in the literature on mental fatigue in sports. We have not aimed to provide a systematic or exhaustive quantitative synthesis but rather to offer a thematic exploration that highlights the evolution of understanding and identifies areas for future research

## Figures and Tables

**Table 1:** Several explanatory theories of MF generation.

Interpretation Theory of MF	Causes of MF	Theoretical Shortcomings
Motivational control theory	Effects of effort–reward imbalance during task performance, leading to a reduction in persistence of performance	Neither the motivationally driven brain regions nor the subjective experience of MF is affected by TOT
Underload theory	Prolonged performance of cognitive tasks may produce experiences of boredom, leading to negative sensorimotor qualities, reduced task engagement and increased task-irrelevant thoughts, leading to decreased cognitive performance	Negative correlation between DMN activity and subjective fatigue
Resource depletion theory	Depletion of available cognitive resources due to continuous allocation of attention to tasks	The rigor of the statistical base is open to question. Disagreement about the cognitive resources accounting for the TOT depletion
Neurotoxic waste disposal hypothesis	Continued use of cognitive resources causes accumulation of toxic waste in neural tissue	Lack of sufficient experimental data to support
